# Women Who Perform Social Egg Freezing as Moral Pioneers: The Case of Ultra-Orthodox Communities in Israel

**DOI:** 10.1007/s10943-024-02062-z

**Published:** 2024-05-23

**Authors:** Maya Maor, Miriam Billig

**Affiliations:** Department of Sociology and Anthropology, Ariel University, Ariel, Israel

**Keywords:** Social egg freezing, Ultra-orthodox, Reproductive technology, Social change

## Abstract

Social egg freezing (SEF) is a new reproductive technology that is increasingly used within ultra-Orthodox Jewish communities, stirring tensions between tradition and modernity. Based on in-depth semi-structured interviews, this study examined how ultra-Orthodox singles who employ SEF engage in social negotiations over gender- and body-related norms. Findings show that participants successfully assimilated SEF by establishing facts on the ground and discreetly spreading information while actively avoiding tensions that may threaten religious tradition. SEF did not push participants into modern individualism or dissolve their strong connection to the community. However they did modify social boundaries and articulated social criticism.

## Introduction

Social egg freezing (SEF) is a new reproductive technology gaining popularity in the Western world, due to the rising age of marriage and rates of women’s education and employment, leading to delayed age of childbearing (Martin, 2010; Rimon-Zarfaty & Schicktanz, 2022). When first introduced in the late 1980s, egg freezing (cryopreservation of oocytes) was offered as an experimental treatment to young women undergoing medical treatments that might damage fertility (Johnston et al., 2022; Rimon-Zarfaty et al., 2021). The initial success of the procedure led to the approval of egg freezing for healthy women facing age-related fertility decline due to so-called “social” reasons – social egg freezing (SEF) (Inhorn et al., 2018; Rimon-Zarfaty et al., 2021). Social motivations for egg freezing are primarily relationship factors (e.g., singlehood), followed by economic factors or some other reason for delaying motherhood (Birenbaum-Carmeli et al., 2021; Inhorn et al., 2018; Kanters et al., 2022).

Albeit central to regulation and funding, the distinction between “medical” egg freezing (MEF) and SEF is highly debated: the desire to conceive while facing age-related fertility decline can be considered a medical condition justifying the use of medical means (Rimon-Zarfaty et al., 2021). Simultaneously, a cultural hierarchy that subjugates social motivations to medical ones is reflected in funding, regulation, and public opinion, which favor MEF over SEF (Pennings, 2013; Rimon-Zarfaty et al., 2021). Indeed, critics have raised ethical concerns and argued that SEF is a tool for medicalizing non-medical social problems (Dondorp and De Wert 2009; Petropanagos et al., 2015).

Social egg freezing raises questions in relation to the tension between tradition and modernity. Such tension is revealed in cultural gender conflicts over the meaning of virginity and modesty, the age and purpose of marriage, and women’s autonomy and control over their bodies, particularly in religiously observant societies. Ultra-Orthodox communities attract significant public attention as they stand at the crossroads between religious piety and conservatism, and rapid social and cultural change (Hakak, 2009; Hitman, 2022; Teman & Ivry, 2021). The ultra-Orthodox comprise 13.3% of Israel’s total population, and with the highest growth rate in developed countries (4%), they are expected to comprise 16% by 2030 (Malach & Cahaner, 2022). Their communities are characterized by high fertility rates, young average age at marriage (22 for women), young age at birth of first child (23), and a large number of children per family (6.1) (Malach & Cahaner, 2022; Regev, 2017). The ultra-Orthodox community in Israel is comprised of three religious’ mainstreams: Hassidic (35.6%), Sephardic (35.3%), and Lithuanian (29.1%) (Regev, 2017). The three streams diverge in their dress codes, importance they attribute to religious studies, types of employment and openness to external influences (Regev, 2017). The ultra-Orthodox tend to segregate geographically and culturally and as a whole reject external influences. Furthermore, ultra-Orthodox communities’ attitudes toward technology, especially digital media, are complex and tend to be negative, especially in regard to the inclusion of children and young people (Rosenberg et al., 2023).

Most ultra-Orthodox parents are heavily involved in partner selection and use the services of local professional matchmakers (Yacovson et al., 2020). Unmarried women of advanced age are considered “problematic to marriage” and may lead to a marriage queue as siblings are expected to marry in the order of their birth. These factors attract severe social pressures to marry from family, peers, and the community. Prolonged singlehood among religious ultra-Orthodox women is connected to increased loneliness, social disconnection, and sexual deprivation (Engelberg, 2016; Yacovson et al., 2020).

Notwithstanding these conservative characteristics, ultra-Orthodox society is exposed to external influences that are expressed in rising education and employment rates among women, which in turn, can also postpone the age of marriage (Malach & Cahaner, 2022). These trends are creating greater disposable income, leisure culture, and the emergence of modern discourse among the growing middle class in ultra-Orthodox society (Malach & Cahaner, 2022). Indeed, a survey from 2017 reveals a significant rise in the age of marriage: while 61% of ultra-Orthodox men aged 25 were married in 2005, only 44% were married in 2016. Similarly, while 80% of ultra-Orthodox women aged 20–29 were married a decade ago, today only 66% of women of these ages are married (Malach et al., 2017). Given the clash between increasing rates of late-singlehood and the socio-religious expectation to have large families, Jewish religious communities and rabbinical leaders have defined growing rates of singlehood as a severe social problem (Engelberg, 2016; Yacovson et al., 2020).

There is scattered and preliminary evidence that ultra-Orthodox communities may coopt/embrace SEF as a technological solution to these tensions (Rimon‐Zarfaty & Schicktanz, 2022; Inhurn et al., 2018). Nevertheless, the ability of ultra-Orthodox women to exert agency in relation to reproduction has been examined only among married women undergoing multiple pregnancies, fertility treatments or prenatal diagnostics (e.g., Teman & Ivry, 2021; Teman et al., 2011; Teman et al., 2016). However, the ability of ultra-Orthodox singles to exert reproductive agency, without explicitly violating social norms, remains understudied.

Social egg freezing can provide a technological solution for late singlehood and the fulfillment of the desire for large families. The present study will address ultra-Orthodox single women who opt for SEF as “moral pioneers”. The term “moral pioneers” refers to the first group in a specific society ARTs and is thereby positioned at the frontier of legal and social change. It was originally used to refer to pregnant women using amniocentesis in the 1980s when it was experimental (Rapp, 1987). Not only are these women the first in their ultra-Orthodox community to use ARTs as single women but they are negotiating perceptions, behaviors, and norms around motherhood, families, and reproduction. The study examines the ways in which single ultra-Orthodox women negotiate with their communities over norms related to the body and gender, tradition and modernity, autonomy and control.

### Literature Review

The relationship between the introduction of ARTs to different cultures and communities and the facilitation of social change with respect to gender roles, family structures, relatedness, and kinship is highly debated. Some scholars argue that dominant notions of biology, gender, and kinship are deeply inscribed in new reproductive technologies and are therefore reinforced by them (Thompson, 2005). For example, Nordqvist (2017) found that even when lesbian families create children through the use of donors, they employ “genetic thinking”, which reproduces conventional families along a set of accepted beliefs. Nonetheless, other scholars have shown that some uses of ARTs, such as single mothers by choice who use both sperm and egg donation, creatively change and challenge traditional definitions of motherhood and kinship, for example, by “maternal bricolage” which relates to practices of “crafting embryos and then finding homes for the ones they do not use” (Hertz, 2021). Similarly, Payne (2016, p. 483) writes that as a result of ARTs, we are able to not only distinguish between biological and social motherhood but also further divide biological motherhood into various “modalities,” such as gestational, genetic, epigenetic, and mitochondrial motherhood.

Marcia Inhorn (2020) outlines seven major global trajectories that signal future potentials that have yet to be realized in the field of ART-based social research, each corresponding to a recent specific influential technological or social development: 1) the profusion of technological innovations; 2) the influence on notions of masculinity as men increasingly become direct users; 3) the interaction with globalized stratification; 4) increasing employment beyond national borders; 5) IVF-based reprogenetic technologies; 6) the interaction of religious sensibilities with ARTs; and 7) the prolonging of reproductive lifespans by employing cryopreservation technologies. The present study focuses on the intersection of at least two of these trajectories and examines how the introduction of new cryopreservation technologies interacts with religious and moral sensibilities among ultra-Orthodox Jews in Israel.

Indeed, demographic trends in industrialized countries, such as an increase in women’s employment and education, a rise in the age of marriage, and a growing awareness of the dramatic decline in female fertility with age, have led to exponential growth in SEF (Johnston et al., 2022). Culture influences women’s motivations to opt for SEF and the expectations and meanings they ascribe to it. For example, a preliminary analysis found that while some motivations for SEF were common for both German and Israeli users (‘waiting for a partner’), some motivations characterized only German users (‘postponing motherhood’) and some characterized only Israeli users (‘planning of and hope for multiple children’) (Rimon-Zarfaty & Schicktanz, 2022). Moreover, since SEF works to synchronize women’s biological and social temporalities, it reflects moral and normative assumptions concerning ‘appropriate’ timetables, the biological clock, motherhood and family, reproductive control, and autonomy (Baldwin, 2019; Rimon-Zarfaty & Schicktanz, 2022; Rimon-Zarfaty et al., 2021). However, unlike other biotechnological innovations, such as human genome research (O’Doherty et al., 2021), organ donation (Ward et al., 2018), and surrogacy (Narh et al., 2021), the influence of SEF on specific societies and social change is yet to be studied.

Like other industrialized societies, in recent decades there has been a marked rise in women’s education and employment as well as in the age of marriage in Israel. However, with a total fertility rate of 3.17 children per Jewish woman, Israel has a significantly higher birth rate than other Western countries, roughly twice the European average (Birenbaum-Carmeli et al., 2021; Israeli Central Bureau of Statistics, 2021). Indeed, Israel is exceptionally pro-natalist; large families are heavily endorsed at the personal, social, and even national level (Birenbaum-Carmeli, 2016; Hashiloni-Dolev & Triger, 2020). Thus, it is not surprising that Israel is a leading force in the development and employment of ARTs; approximately 6% of all babies born in Israel were conceived via in-vitro fertilization (IVF) (Ministry of Health, 2023). This trend is also manifested in the fact that Israel was the first country (with the U.S.) to approve SEF for healthy women. Approximately 10,000 cycles of SEF have been performed in Israel since 2011; the number of SEF cycles per year increased significantly from less than 100 in 2012 to more than 800 in 2019, and the numbers continue to rise (Koch-Davidovitch, 2021).^1^ Affording SEF remains a significant hurdle for many women, mirroring the situation in various industrialized nations such as the UK, the Netherlands, the US, Australia, and Korea, where Social Egg Freezing (SEF) is typically self-funded (Birenbaum-Carmeli, 2023). Nonetheless, in Israel, SEF costs have shown a recent decline, currently ranging from $1400 to $5000 per cycle—markedly less expensive than in the US (approximately $10,000) and other industrialized nations (Birenbaum-Carmeli, 2023). Moreover, Meuhedet, the third-largest Health Maintenance Organization (HMO) in Israel, which caters to extensive ultra-Orthodox communities (Ziv-Baran, 2023), partially subsidizes SEF. These reduced expenses, particularly for members of Meuhedet HMO, may serve as an encouraging signal to ultra-Orthodox women.

### SEF in Religious Communities

Unlike most forms of ARTs, SEF is generally used by single women, raising moral controversies among different religious communities. The general acceptance of SEF by Protestant Christianity contrasts with Catholicism, which forbids all forms of reproductive technology—because all are deemed “unnatural,” whether they are intended to prevent or to assist reproduction (Inhorn et al., 2020; Czarnecki, 2015).

While some Islamic permit egg freezing by single women (Inhorn et al., 2020), other Islamic fatwas object to SEF as they fear it may create a strong expectation among single women to use their frozen eggs one day, regardless of whether they eventually marry (Chin & Saifuddeen, 2022). In Malaysia, for example, there is a blanket ban on single Muslim women freezing their unfertilized eggs to be used later in marriage. According to the fatwas, women’s eggs that were frozen when they were single cannot be used even if they marry in the future since they represent their “unmarried body and self” (Chin & Saifuddeen, 2022). Only married Muslim women with valid medical reasons are allowed to freeze their eggs, and even then the eggs can only be fertilized by their legally married husbands (and not after divorce or death of the husband) (Chin & Saifuddeen, 2022).

Many religions consider virginity a precondition for marriage, where the hymen provides the physical proof of virginity. For example, according to Jewish Halacha (religious law) (Leviticus 15, 25–28), single Jewish women preserve their hymen intact until their wedding night. Thus, another factor that raises religious concerns is related to the damage to the hymen during egg collection. In the 1980s egg collection was done abdominally through laparoscopy. Subsequently, a more progressive, less invasive, and complicated vaginal approach was developed and is now medically accepted. In Islamic Turkey, to appease parents, physicians use the abdominal approach. In contrast, Israeli medical policy is to use the vaginal approach. Technically, women lose their virginity through vaginal egg collection, although from a halachic point of view only the removal of the hymen by sexual contact is prohibited (Nahari, 2008).

Modern Judaism is characterized by strong pro-natalist tendencies, and a high birthrate is considered a significant expression of religious observance (Teman et al., 2021). Studies show that religious Jewish families, even ultra-Orthodox, embrace the use of fertility-enhancing ARTs, with cultural adaptations, while remaining ambivalent toward other forms of ARTs such as prenatal testing (Teman et al., 2011). Considering the rise in the age of marriage (Malach et al., 2017), SEF can benefit the ultra-Orthodox Jewish community by prolonging the potential of genetic motherhood. Nevertheless, the relative acceptance of ARTs in ultra-Orthodox communities in Israel is mainly reserved for married couples, while practices related to the fertility of singles remain taboo. At the same time, social change is slowly in the making: behind closed doors there is a willingness by some Rabbis to accept SEF as a solution for the growing number of singles of advanced age. However, even Rabbis who permit SEF to individual women who turn to them maintain public silence on the subject (Billig & Maor, 2024).

Furthermore, the use of SEF raises distinct ethical issues among the ultra-Orthodox. Social egg freezing can thus be theorized as a “techno medical fix” (Martin, 2010) for social problems lying well beyond Jewish women’s individual control, such as age-related fertility decline (Birenbaum-Carmeli et al., 2021). In contrast to their secular counterparts, ultra-Orthodox singles are often expected to occupy a status of ‘disembodied embodiment’: refraining from expressing their sexuality or fertility and minimizing the place of their body (Inhorn et al., 2020). Social egg freezing can offer a technological bypass, enabling singles to experience their embodiment and fertility without violating social norms. For example, Inhorn et al. (2020) described how one Jewish religious interviewee who had a strong reaction to the hormonal stimulation and produced many eggs experienced SEF as positive and empowering and as allowing her to connect to her sexuality and fertility. Social egg freezing can offer a technological solution for other distinct social problems as well. In some ultra-Orthodox communities, women find it difficult to find a partner because they tend to be more educated and professionally experienced than their male counterparts (Inhorn et al., 2020). In such cases, SEF may provide leverage to find an adequate partner. Social egg freezing can also help ultra-Orthodox women stuck in a “marriage queue” as an older sibling remains single or separated women waiting for their husband’s permission to divorce.

### The Aims of the Study

The present study will explore how ultra-Orthodox singles who employ SEF engage in social negotiations over gender- and body-related norms in three distinct areas:

1. Control of information: drawing new boundaries about legitimate knowledge
2. The relationship between the single women in advanced ages and the community
3. Personal empowerment and the development of social criticism

## Methods

To recruit participants for our study, we began with personal acquaintances, which led to snowball sampling. In addition, we produced an electronic flyer and asked participants to share it in relevant social media and mailing lists following their interview.

The two authors interviewed the participants together, in Hebrew, mostly via online meetings, in response to interviewees’ preferences. This preference allowed us to maximize privacy and save logistic resources, such as time, removing participants from their natural surroundings, and travel costs. The authors conducted the interview together as each has different professional and personal insights on the subject: Author 1 is familiar with medical sociology and the study of SEF while Author 2 specializes in the study of religious communities and is familiar with the ultra-Orthodox community in Israel.

The interviews were semi-structured, beginning with an open-ended part that was conducted as a free conversation, in which the authors disclosed their connection to the research subject and professional experience. Total confidentiality was guaranteed. After trust was gained, we asked participants to freely tell us about themselves and their family’s involvement in the process of SEF. Then we moved the conversation to their experience of SEF. Participants were asked to describe their prior knowledge of the body and fertility; their initial decision to undergo SEF; their personal experiences of SEF, sources of support, and barriers. We specifically asked about communication with educators and rabbinic authorities and the role of faith in their decision and experience of SEF. Participants were encouraged to disclose any relevant information regarding the attitudes of their community, family, and peers toward SEF in general and toward their own involvement in the process.

In-depth interviews were conducted in Israel during the winter of 2022 (December 2022–February 2023) with ultra-Orthodox women aged 31–43. Due to prominent pro-natalism in ultra-Orthodox communities and social norms of marriage at a young age, most participants reported beginning to consider SEF starting from their mid-20s. As Israeli regulations permit SEF between ages of 31 and 40, most reported undergoing the procedure between ages of 31 and 35, with a mean age of 33. The participants’ mean age is younger than the mean age for SEF in contemporary Western societies, which is 38 (Varlas et al., 2021; Kastani et al., 2024; Hirsch et al., 2024). In contrast to IVF, most SEF users do not have a fertility problem. Success rates depend on age at the time of eggs collection, which is considered ideal before age 35 (Varlas et al., 2021). Therefore, participants of the present study were likely to have good outcomes due to their relatively young age and indeed most reported overall satisfaction from the number of oocytes frozen at each cycle.

All interviews conducted as part of this study were long and complex, ranging from one to three hours. The authors read the verbatim transcripts and coded them for emergent themes using the grounded theory approach (Smith, 2003). The study received the approval of the University’s Ethics Committee and all participants signed an informed consent form. All the names mentioned in this paper are pseudonyms. Participants were informed that they could stop the interview at any stage. Nearly all the women requested that the names of any rabbis they mentioned remain anonymous.

## Results

This research is the first systematic, in-depth sociological inquiry of a sample of ultra-Orthodox women who have undergone SEF. Studying this group enables us to understand the specific culturally modified enactment of a new reproductive technology. Concurrently, the adoption of advanced medical technologies involves complex, shifting negotiations of autonomy and control between women and their communities.

The women participating in this study identified as ultra-Orthodox from three general ultra-Orthodox sectors: Lithuanian, Hasidic, and Sephardi (Table 1).

**Table 1:** Participants characteristics

Participant number	Pseudonym	Age	Stream	Location	Profession	Education/equivalent
1	Gita	38	Lithuanian	Bnei-Brak	Gynecologist	MD
2	Sara	41	Lishuanian	Jerusalem	Kindergarten teacher	MA
3	Yochi	34	Hassidic	Jerusalem	Public relations	BA
4	Fruma	37	Lithuanian	Jerusalem	Kindergarten teacher	MA
5	Malka	33	Lithuanian	Jerusalem	Social Work	BA
6	Rachel	31	Hassidic	Bnei-Brak	Education	MA
7	Frida	34	Lithuanian	Jerusalem	Event organizer	Certification Studies
8	Naomi	32	Lithuanian	Ariel	Education	MA
9	Lea	34	Sfaradic	Bnei-Brak	Special education	BA
10	Yehudit	37	Sfaradic	Jerusalem	Education	BA
11	Yafa	32	Lithuanian	Bnei-Brak	Secretary	Certification Studies
12	Bat- Sheva	35	Hassidic	Bnei-Brak	Education	BA

Most of the women worked, commonly in education and services, and most were from the middle social class. Most participants came from large families, and most had married siblings. Half of the participants lived with their parents, and half lived alone or with female roommates.

### Control of Medical and Body-Related Knowledge

When You’re a Bride, We’ll Let You Know

Ultra-Orthodox society is characterized by tight top-down regulation of information in general, particularly in relation to digital media, to “protect” the community from exposure to postmodern ideas promoting individualism and from progressive ideas concerning gender roles and sexuality, to preserve traditional values and practices. This tight regulation is expressed through the enforcement of gender separation throughout all domains of social life, norms of modesty in dress, and controlling discourses of sexuality and the body (Nahari, 2008). In general, internet, social media, television, and smartphones are forbidden (Engelberg, 2016). This exacerbates ultra-Orthodox women’s lack of knowledge about sexuality and women’s reproduction, which is not uncommon in contemporary Western societies (Saral et al., 2023).

To preserve modesty, strict gender separation in all social life domains begins at the age of three and is increasingly enforced. Education is based on traditional family values and marriage at a young age is heavily endorsed. In this context, singles over the age of 25 are stigmatized as “old singles”; their social status declines, and they are pushed to the fringe of the community. Many participants reported feelings of loneliness before starting the SEF process due to the sense of a growing distance between them and their married friends who conform to social timetables. According to participants, a significant cause of the growing distance was a lack of common experiences and conversation topics, which increased their sense of loneliness and distance.

Before the wedding, brides meet with bridal instructors who provide information about sexual intercourse and their roles as wives and mothers. Consequently, girls and women who have yet to marry are only exposed to partial, limited sexual and physical education. As Sara (P2), 41, from Jerusalem, describes:

Everything about sexual education is secret; you don’t know your body and sexuality. We are told to marry as early as possible to build a big family, but nothing more… and the bridal instructor will tell you everything before the wedding.

Social egg freezing revolves around discourses of body, sexuality, and fertility and is therefore considered taboo in ultra-Orthodox society. Many interviewees felt hurt and angry over what they experienced as they were deprived of important information. For example, Malka (P5), 33, from Jerusalem, expressly referred to the lack of information regarding the effect of age on fertility and the biological clock. This information is paradoxically most relevant to those who do not receive it because they have yet to marry. As Sara (P2) relates:

There wasn’t any talk about gynecological issues, hormonal profiles, or painful issues… No one talked with us about the menstrual cycle or low ovarian reserve… We lack reassuring information about the body; even the family physician didn’t talk to us about it.

Left in the dark, the women felt confused and helpless at the beginning and needed a guiding hand, as explained by Malka (P5):

The most challenging phase was how to turn to someone—because I didn’t know whom to turn to. I needed someone to organize what I needed to do; it was something I needed and didn’t have. I could not mobilize myself… until a friend of mine made some calls and told me what to do and where to go. I didn’t even know a gynecologist... I didn’t know enough to know if SEF suits me Halacha-wise, I didn’t even know if it’s allowed or forbidden.

In contemporary Western societies, a gynecological examination can be a significant source of body-related knowledge and experiences (Eid et al., 2019). In contrast to other social groups in Israel, in ultra-Orthodox circles, it is not socially appropriate for women to undergo a gynecologist examination before their marriage, regardless of their education. Because SEF necessitates gynecological examinations, participants obtain new forms of knowledge and gap reduction.

Getting to know other women in similar situations with whom to share experiences and information helped participants. Behind the framing of ‘body-related knowledge’ as irrelevant to single women, stands the religious-traditional belief that fertility is only legitimate in the context of marriage. By nevertheless obtaining information, participants draw new social boundaries. According to these boundaries, body-related knowledge becomes relevant to their lives even though they are unmarried. They challenge their dependency upon the authority of the community for information and thereby serve as moral pioneers who challenge social boundaries and taboos.

### Gathering Information and Choosing to use SEF

“I’m not only living in dreams—I’m taking action.”

Participants actively searched and obtained the information needed to start the process independently. As written above, among ultra-Orthodox communities, access to the Internet is tightly regulated to avoid exposure to external influences. At the same time, ultra-Orthodox women increasingly participate in the higher education and employment market (Malach & Cahaner, 2022). Some participants gathered information by surfing the internet at the college library. Others received information from colleagues and acquaintances from outside ultra-Orthodox circles, e.g.: national-religious or even secular ones, in workplaces that cater the general population mostly in the education sector. Some of the relevant information reached participants only after they were already engaged in the first stages of the process. After initiating SEF, some medical information was obtained from medical personnel. Some participants became informal volunteers who provided help varying from technicalities (e.g. how to properly inject oneself with IVF medications) to psychological and moral support.

Most interviewees described a moment of clarity in which they became aware that their advanced age necessitated action. The painful aspects of the resolution confronted participants with the decision of who to confide with. Many chose not to disclose going through SEF to their mothers or other family members in order not to hurt them by reminding them that their “biological clock is ticking”. Others emphasized their emotions as individuals, as Sara (P2) recounts: “It was my birthday, and I started the SEF process. It was a sort of gift to me.” Lea (P9) described the process reaching the decision to embark on SEF follows:

I don’t want to cry at the age of 36, saying why hadn’t I done it back then—because I fantasized a guy would come. “I’m not only living in dreams and imaginations but also taking action, because I want to be wise and not be upset with myself later.

Contrary to what is expected of them as unmarried women, participants challenged the social norms in their community by gathering information about their bodies, reinforcing their feelings of independence. Nonetheless, at the same time, participants also reaffirm accepted social boundaries by preserving codes of modesty and refraining from romantic/sexual relationships with men. Furthermore, none of the participants chose to freeze embryos via sperm donation, because they knew their communities’ fierce objection to single motherhood by choice.

### SEF as a Tool for Improving Self-Esteem/Benefiting the Self

“SEF will improve my opening point ״

From a religious perspective, SEF allows single women to extend and increase their childbearing potential without violating the moral norms of celibacy before marriage. In this way, SEF becomes a new medical and technological means to express "effort" [in Hebrew: hishtadlut] that is, striving or taking action to fulfill a religious ideal, to fulfill social and religious expectations for a large family even if they marry at a relatively older age. As Lea (P9) describes: “She told me: God gives you means, like, today you’re young so your eggs are good, and at least you preserve that right with your husband, in God’s will.” Rachel (P6) adds:

The ultra-Orthodox say all sorts of things, such as when you finish [with SEF] your groom will come. To take an action that is blessed. After I started the process of SEF I prayed: “God, observe my will to build a family and see how I’m hereby ‘taking action’.

Using SEF to improve one’s status also rose in relation to matchmakers' preliminary references to SEF. While still not common, some participants described how SEF begins to emerge in intimate conversations with the matchmakers, as Yochi (P3) explains:

One day, the Matchmaker asked whether I had undergone SEF, and she explained that it might increase my chances … She said if my age deters a guy, telling him I’ve done SEF could improve my chances. This question made me happy; it was a sign that I had done the right thing.

Social egg freezing may also decrease the stress and pressures participants felt they face to compromise in spouse selection to guarantee genetic motherhood, as Yochi (P3) states:

In the past, having a family was a precondition to existing in the world… Today, the world has become mor prosperous with possibilities… I don’t want to compromise… I always tell people that I’m picky… In the past I didn’t have the option to be single even at the cost of a bad relationship… My grandma was horrified to hear that I’m freezing eggs… she would like me to marry someone, maybe it won’t be “wow” but I should do it.

Participants’ experiences of using SEF to improve their status echoes theorization of egg freezing as “promissory capital”. In a social climate valorizing reproduction and large families, SEF can be theorized as preserving fertility and the sense of personhood and self-esteem. In this sense, SEF, particularly when defined and explained to others, is a form of social capital (Birenbaum-Carmeli et al., 2021). Because SEF represents a scientific effort to manage the future, the frozen eggs themselves can be considered a form of “promissory capital” (Lemke, 2019; Rimon-Zarfaty & Schicktanz, 2022; Thompson, 2005).

### Articulating Social Criticism

“Regaining Control Over My Body ״

An increased sense of autonomy following the SEF experience significantly transformed participants by: 1) catalyzing changes in self-image and esteem; 2) leading them to develop a more critical approach toward their community; 3) opening them up to new ideas about family, gender roles, etc.

Coping with the challenges that arose during the SEF process empowered participants and increased their sense of competence and self-confidence, as Rachel (P6) described: “I had never undergone any surgical procedure, or lost my virginity… these things were extremely difficult for me, and I decided to undergo SEF regardless.”

Malka (P5) explained that SEF serves as an existential crisis and that coping with it made her feel stronger and more capable:

SEF confronts you with being alone and with your singlehood; it’s a mirror to where your life is. You’re standing in front of yourself for the first time and coping. If you don’t act now, you won’t have anything left; it was there that I realized I’m not young anymore, what I feared has come true and now I have to deal with it face to face.

The boost in self-confidence is stark in relation to Fruma (P4):

It is indeed an invasive procedure, it’s unpleasant, but it’s under my control. Regaining control over my body… My therapeutic goal is not to always be in this stress!

Lea (P9) explained the rationale behind the decision to undergo SEF in a conversation with a friend who supported her: “She told me it’s a new thing out there, so why not use it?” Lea’s words demonstrate the transition from viewing medical technological innovation as a positive opportunity for self-efficacy rather than as a threat.

Discussing the new possibilities involved in SEF and the experiences that come with the process encouraged participants to develop a more critical approach toward their social position. Rachel (P6) has become more conscious of her body and criticizes the ultra-Orthodox community while remaining optimistic about the future:

Everything concerning sexuality is very partial; we lack acquaintance and shy away from asking questions about our body. Wanting to know is forbidden… I’m sure it will get better at some point in the future. The ultra-Orthodox sector is a couple of years behind.

Gita (P1), a gynecologist who treats religious women suffering age-related fertility loss, criticizes the ultra-Orthodox establishment for impeding access to information about SEF:

Rabbis and spiritual leaders need to speak up; it doesn’t make sense to say these things only behind closed doors, as well-kept secrets—they need to be said out loud, publicly. I want someone that begins the process not to think that she needs to hide the process and be ashamed of it. She needs to know that this is the appropriate thing to do.

Sara (P2) articulates another criticism, directing it at men who want to date women much younger than them:

I want to throw up on them. He doesn’t want me. He’s looking for someone up to age 38 and I’m 41. From my point of view, children should be the product of a relationship and not its purpose. I’m not just as “a surrogate” mother… Men live in the myth that women have an “expiry” tag on their fertility while they can have children until the age of 120.

Sara (P2) and Gita challenge common social norms in ultra-Orthodox communities about two distinct issues: censoring crucial body-related information and double social standards regarding gender and age in the marriage market. Inherent to both criticisms is the refusal to accept present gender power relations and the use of their perspective as women as a valid vantage point from which to articulate a more just social order. This challenge also manifests in the “holy grail” of the ultra-Orthodox community: the ideal of the traditional family.

Undergoing SEF also exposed participants to new approaches and ideas regarding the ideal family. Lea (P9) describes a conversation she had with a friend from outside the ultra-Orthodox community, who accompanies her in the process of SEF:

She told me: ‘Lea I can see that you’ll be an amazing mom… Think about it… your children won’t be miserable without a father, you have a lot of good things to offer’. I was shocked… It brought tears to my eyes… Opened my head… I want to be a young mother… It’s reassuring to know that if I won’t find my guy until I’m 36 I could be a single mother]. But to do so in an ultra-Orthodox community is unthinkable!! People would think I had been with a guy outside of marriage… I must open my head in this process by myself. We need to talk to educators… to stimulate them.

Rachel (P6) also dares to consider the possibility of single motherhood:

I don’t know if I will be able to marry, I don’t want to lose the privilege of motherhood. I love children and I really feel the urge to have children by myself. But my conscious still won’t let me do it.

As we have seen, through the process of SEF, participants challenge social norms and religious prohibitions. In addition, they begin to articulate criticisms about their social stigmatization and even think about alternative social scripts, such as the possibility of single motherhood.

## Discussion

Our study demonstrates that exposure to the medical technological innovation of SEF encouraged single ultra-Orthodox women to search for information previously denied from them since it was limited only to married women. By obtaining this knowledge, they expanded the social boundaries of legitimate body-related knowledge. Their exposure to SEF-related information led them to express more independence in making decisions regarding their body and reproductive potential, often without any support or even knowledge from their families.

Subsequently, these ultra-Orthodox singles led negotiations that challenged core values and social norms related to family structure and gender roles in ultra-Orthodox society. Unintentionally, they acted as moral pioneers by negotiating social norms regarding gender and reproduction. The literature often theorizes the adoption of modern practices and social change as being at odds with old religious traditions (Salari et al., 2018; Billig, 2022). Nevertheless, the case of the ultra-Orthodox singles shows that new and old practices can be interwoven in a series of complex, micro-negotiations without favoring any side of the old-new dichotomy. The use of SEF technology, with its subsequent necessary social transgressions, was made to strengthen membership of the religious community. The relationship between the singles and the community did not dissolve but rather took on a different, more critical form.

The sophisticated relationship between modernity and tradition is demonstrated in at least four crucial points in participants’ journey: (1) the social transgressions entailed in the SEF process serve as a toll in the effort to participate in the religious ideal of bearing as many children as possible without violating the moral norms of modesty and celibacy before marriage; (2) participants refrained from disclosure to family members or rabbis they suspected would oppose the process, confiding only with those they thought would provide support, and thereby abstaining from unnecessarily stirring up controversy; (3) most participants continued to harness their best efforts to find a marriage partner throughout the SEF process, thus proving their loyalty to building a traditional family; and (4) limiting SEF technology by choosing not to freeze embryos made with sperm donation, despite its proven benefits (Varlas et al., 2021). In addition, participants did not publicly or openly discuss the option of single motherhood, thereby preserving the traditional ideal family.

In contrast to neo-liberal risk society that conceptualizes SEF as an individual form of pro-active assumption of responsibility for risks associated with reproduction (De Proost & Paton, 2022), the traditional Jewish context envisions SEF as a technological tool to build large families even in late marriages. Thus, SEF can be experienced as religious obligatory effort – ‘hishtadlut’, as studied by Teman et al. (2016) in relation to other reproductive choices. Specifically in the Jewish community, SEF can be viewed as a technological solution to the decrease in social status of singles as their age advances and reduces their chances of bearing many children. If SEF becomes more normative in ultra-Orthodox communities, it could be used as means of maximizing self-worth in the marriage market.

Many of the feelings and experiences reported by participants, such as self-empowerment and hope mixed with loneliness and regret, and decreased pressure to find a partner—are common to SEF users in other socio-cultural contexts (e.g. Baldwin et al., 2019; Hathway, 2022). The distinctiveness of ultra-Orthodox context is that merely initiating SEF necessitates negotiation of social boundaries. This leads participants to discover new abilities, boosts their self-esteem and may encourage some of them to aspire to change their social position within the community.

## Limitations

This study has several limitations. Future studies including greater numbers of participants could further elaborate on differences between women who freeze eggs from the three main ultra-Orthodox streams. The study focused only on the viewpoint of women who perform SEF. Future studies should include other interested parties: Rabbis, community leaders, and organizations. Follow-up studies could be large-scale attitude surveys disseminated to the entire ultra-Orthodox community, including men and ultra-Orthodox women who choose not to freeze eggs. Similarly, long-term studies would be able to determine whether SEF encourages single motherhood among ultra-Orthodox communities.

## Conclusions

Ultra-Orthodox singles successfully assimilated SEF by establishing facts on the ground and discreetly spreading information by word of mouth while actively avoiding tensions that may threaten religious tradition. Their use of SEF did not push women into modern individualism or dissolve their strong connection to tradition and the community. However, as moral pioneers, they did modify social boundaries and articulated social criticism. On the other hand, they maintained their religious faith and refrained from “pushing too much”, e.g. not considering single motherhood by choice. Social transgressions were made for the long-term preservation of the ideal of the traditional family. Understanding the social implications of SEF in ultra-Orthodox communities is important for devising patient-centered fertility care and medical-educational resources to address the lack of information among women and medical professionals in the field of fertility. Our findings demonstrate a need for family practitioners to provide relevant fertility information to young women from traditional communities who do not visit gynecologists before marriage.

## Footnotes

1 For a discussion of various aspects of social egg freezing in Israel in general see (Birenbaum-Carmeli, 2023).
